# Rehabilitation exercise assessment using inertial sensors: a cross-sectional analytical study

**DOI:** 10.1186/1743-0003-11-158

**Published:** 2014-11-27

**Authors:** Oonagh M Giggins, Kevin T Sweeney, Brian Caulfield

**Affiliations:** School of Public Health, Physiotherapy and Population Science, University College Dublin, Dublin, Ireland; INSIGHT, University College Dublin, Dublin, Ireland

**Keywords:** Inertial sensors, Rehabilitation, Exercise, Performance classification

## Abstract

**Background:**

Accurate assessments of adherence and exercise performance are required in order to ensure that patients adhere to and perform their rehabilitation exercises correctly within the home environment. Inertial sensors have previously been advocated as a means of achieving these requirements, by using them as an input to an exercise biofeedback system. This research sought to investigate whether inertial sensors, and in particular a single sensor, can accurately classify exercise performance in patients performing lower limb exercises for rehabilitation purposes.

**Methods:**

Fifty-eight participants (19 male, 39 female, age: 53.9 ± 8.5 years, height: 1.69 ± 0.08 m, weight: 74.3 ± 13.0 kg) performed ten repetitions of seven lower limb exercises (hip abduction, hip flexion, hip extension, knee extension, heel slide, straight leg raise, and inner range quadriceps). Three inertial sensor units, secured to the thigh, shin and foot of the leg being exercised, were used to acquire data during each exercise. Machine learning classification methods were applied to quantify the acquired data.

**Results:**

The classification methods achieved relatively high accuracy at distinguishing between correct and incorrect performance of an exercise using three, two, or one sensor while moderate efficacy scores were also achieved by the classifier when attempting to classify the particular error in exercise performance. Results also illustrated that a reduction in the number of inertial sensor units employed has little effect on the overall efficacy results.

**Conclusion:**

The results revealed that it is possible to classify lower limb exercise performance using inertial sensors with satisfactory levels of accuracy and reducing the number of sensors employed does not reduce the accuracy of the method.

**Electronic supplementary material:**

The online version of this article (doi:10.1186/1743-0003-11-158) contains supplementary material, which is available to authorized users.

## Background

Exercise rehabilitation after a lower limb surgical procedure, such as total hip arthroplasty or total knee arthroplasty or in the treatment of lower limb musculoskeletal conditions such as osteoarthritis (OA), is accepted as standard and essential treatment [1–3]. Traditionally, rehabilitation exercise is delivered in a hospital or clinic environment; however, recent years have witnessed an increasing demand for more efficient health care delivery which has resulted in an increase in home based rehabilitation. However, many patients encounter various difficulties when performing their rehabilitation exercises at home. For instance, without the supervision of their therapist, patients may execute their exercises incorrectly [4]. Incorrect alignment during exercise, incorrect speed of movement and poor quality of movement may have an impact on the efficacy of exercise and may therefore result in a poor outcome [4]. Patient adherence is also a major problem associated with home based rehabilitation exercise. Up to 65% of patients report being non adherent or only partially adherent to their exercise programmes, with over 10% failing to complete their programmes [5]. The degree to which patients adhere to their exercise programme may also influence the success of rehabilitation. Accurate assessments of adherence and exercise performance are therefore required in order to ensure that patients both adhere to and perform their exercises correctly.

Recent research has explored ways in which technology can be used to enhance home exercise by providing feedback and encouragement to patients. Biofeedback systems have been advocated as they can provide important information on exercise technique and accuracy, allowing patients to correct their movements in real-time. They can also provide patients with an incentive to exercise. Electromyography and real-time ultrasound biofeedback systems have shown potential in rehabilitation [6, 7]; however, the expense of these systems and the expertise required to operate them means that they are inappropriate for patient use in the home. Commercial videogames, such as the Nintendo Wii and the Kinect from Microsoft, have recently gained a lot of interest as rehabilitation tools as they are in-expensive alternatives, that can be operated in the home [8–15]. The Nintendo Wii uses a wireless, accelerometer-enabled controller, which the user holds to track their movement, while the Wii Fit uses a balance board to measure movement. These interfaces can only measure gross body movements and are not suitable to track the subtle movements that occur during most rehabilitation exercises. The Kinect consists of an RGB (red-green-blue) camera and a depth sensor, which provide full-body three-dimensional (3D) motion capture and joint tracking capabilities. The accuracy and reliability of the Kinect system at measuring joint angles has been shown to be comparable to motion capture [16]. However, the accuracy of the Kinect decreases as the distance from the camera increases, and it also struggles with occluding body parts or objects in the scene, [17] which is a significant drawback to its use in the home environment.

Inertial sensors have been proposed as a means of tracking movement during exercise and providing information on technique and accuracy. The small size and unobtrusive nature of these sensors makes them an ideal solution to measure movement and therefore deliver feedback to patients as they perform their exercises. In addition, the low cost and the usability of these sensors has resulted in a number of researchers developing inertial sensor-based systems to address the problems associated with home exercise therapy, with commercially available systems, such as Xsens MVN being proposed to aid rehabilitation [18].

Research in this field has evaluated the use of multiple inertial sensors to evaluate exercise quality [19, 20]. In [19], five body worn accelerometers and machine learning classification were used to distinguish correct from incorrect performance of three lower limb exercises performed by healthy college students. More recently, the same group evaluated the use of five sensor nodes and multi-label machine learning classifiers to assess exercise performance in patients with knee OA [20]. However, using multiple sensors to track exercise performance can be overly cumbersome, which may limit the usability of an inertial sensor based biofeedback system. Reducing the number of sensors that are required to deliver biofeedback would not only reduce the cost of the system but make it more user-friendly for patient use in the home. Preliminary research has provided support for the use of a single sensor approach to evaluating exercise performance during a range of rehabilitation exercises [21]. More recent work investigated whether reducing the number of sensors utilised to evaluate exercise performance, from three to two, to one, had a detrimental impact on classification efficacy scores [22]. The results obtained in this study revealed that not only is it possible to classify exercise performance using inertial sensors with reasonably high levels of accuracy, but a single sensor placed on the lower limb can provide sufficient information on exercise performance to accomplish this, removing the requirement for multiple sensor units. While these results provide support for a single sensor approach to exercise performance evaluation and biofeedback, these investigations were performed with young, healthy participants. Therefore further research was required to investigate whether a single inertial sensor can be used to accurately evaluate exercise performance in a clinical cohort.

The objective of this study was to determine whether lower limb exercise can be evaluated in a cohort of patients using data from inertial sensors and machine learning classification methods. This study also sought to investigate whether using a single sensor can provide sufficient information to accurately evaluate and classify exercise performance. This work seeks to provide further evidence to support the use of a single inertial sensor as an input to an exercise biofeedback system.

## Methods

A cross-sectional analytical study was conducted to examine two research questions; 1) whether lower limb exercise performance in a clinical cohort can be accurately classified using a machine learning classifier and data obtained from three inertial sensors, and 2) whether a reduction in the number of sensors used has a significant impact on the classification accuracy scores. The protocol of this study was approved by the Committee of Human Research Ethics in University College Dublin and informed consent was obtained from all study participants.

All data acquisition for this study took place in a local physiotherapy clinic. A sample of convenience of suitable participants was selected for this study from the clinic. The inclusion criteria were; male or female patients who were attending the clinic, aged between forty and eighty years, and who had performed or were performing lower limb exercises for a musculoskeletal or orthopaedic condition or injury. The exclusion criteria were; a lower limb injury that would limit ability to perform the exercises under investigation, poor functional balance or mobility, any other medical condition that would limit ability to participate in exercise, or cognitive or language difficulties. A screening questionnaire was used prior to enrolment to ensure that each participant was suitable for inclusion in this study.

Participants performed ten repetitions of each of the seven lower limb exercises studied. Three of these exercises were performed in standing (hip abduction, hip flexion and hip extension), one exercise was performed in sitting on a standardised chair (knee extension) and three exercises were performed while lying supine on a plinth (heel slide, straight leg raise (SLR), inner range quadriceps (IRQ) using a rolled towel under the knee). These exercises were studied as they are commonly prescribed to patients following a lower limb injury or surgery [22, 23] and are fully described in [21]. Participants were given standardised verbal instructions and a demonstration by the investigator on how to perform each exercise correctly and were allowed a practice trial of each exercise. The exercises were performed using the participants’ affected limb only. Where there was bilateral pathology, as was the case with participants with bilateral knee or hip OA, the exercises were performed using the more affected side, provided the participant was comfortable to do so.

**Figure 1:**
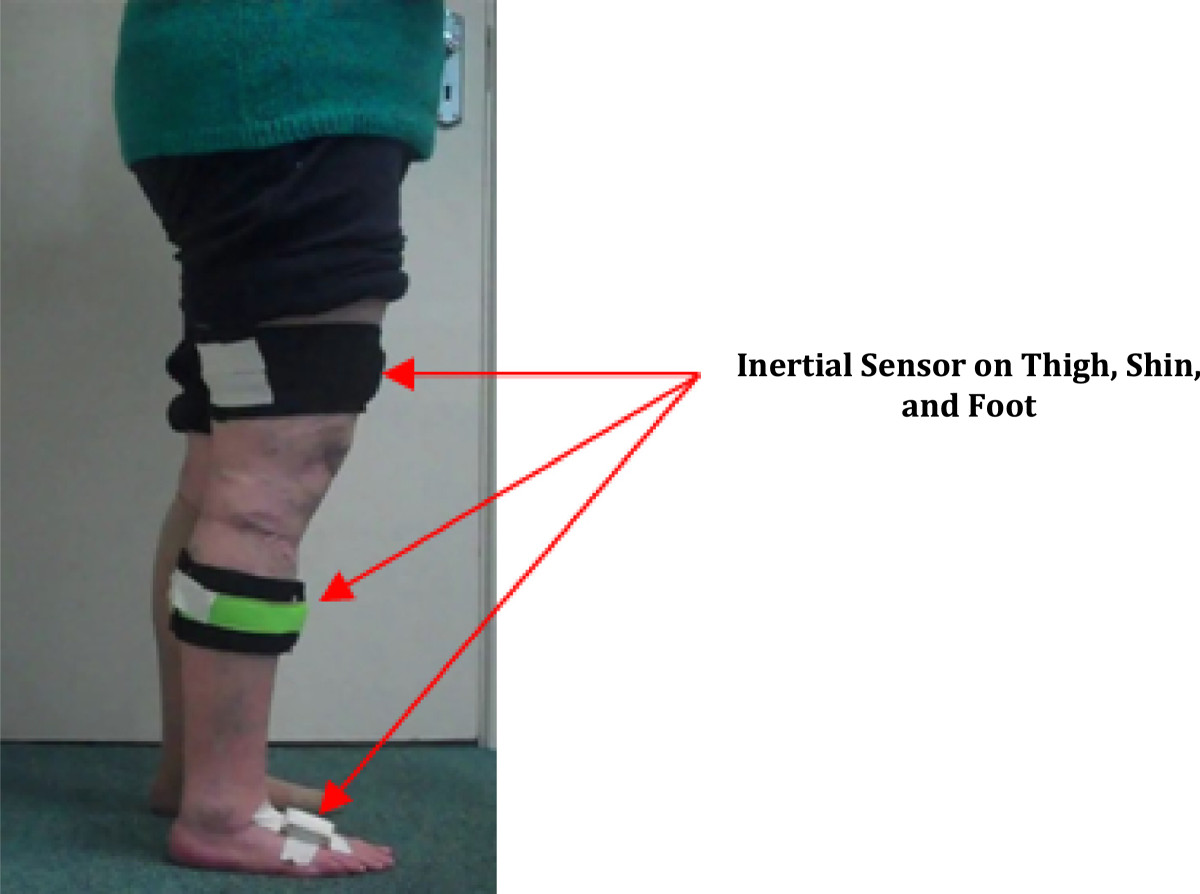
**Inertial sensors secured to thigh, shin and foot.**

Three inertial sensors units (Shimmer, Dublin, Ireland) were secured to the leg that was being exercised for data collection; one on the anterior aspect of the thigh, one on the anterior aspect of the shin and one on the dorsal aspect of the foot (Figure 1). The inertial sensors on the thigh and the shin were secured using a neoprene strap, which contained a pouch to house the sensor, while the foot sensor was secured using athletic tape. The orientation and positioning of each sensor was kept consistent on each participant. Each sensor contained both a tri-axial accelerometer and a tri-axial gyroscope sampling at 102.4 Hz.

The Shimmer 9DOF Calibration Application (Shimmer, Dublin, Ireland) was used to calibrate the accelerometer and gyroscope sensors of each inertial sensor prior to data collection each day. The Multi Shimmer Sync application for Windows (Shimmer, Dublin, Ireland) was used to capture synchronised inertial sensor data over Bluetooth from the three sensors during each of the exercises. The raw inertial sensor data captured were saved onto a computer for off-line post-processing and analysis.

In this study, exercise performance was evaluated and designated as correct or incorrect. A correctly performed exercise is one in which the exercise is executed with correct alignment, speed and quality of movement. If there is an error in one of these features of performance, the exercise is deemed to be incorrect. During data collection, the investigator, who is a physiotherapist, observed each participant as they performed each repetition of each exercise. The investigator rated each repetition of each exercise and labelled the exercises as correct or incorrect. Where an exercise was performed incorrectly, an error label (how the exercise was performed incorrectly) and a severity score on a ten-point scale were given. Where two or more errors occurred during an exercise, the investigator selected the error which had the largest severity measure attributed with it as the label for that exercise. The possible error labels studied for each exercise are outlined in Table 1. Intra-rater reliability was not established in this study.

**Table 1:** **Error labels studied for each exercise**

Exercise	Error Labels
Heel slide	● Heel Lifts – Heel lifts off supporting surface during exercise.
	● Hip ER – External rotation (ER) at the hip joint during the exercise.
	● Jerky Movement – Exercise is performed with a jerky or uncontrolled movement.
Hip abduction	● Hip ER – External rotation (ER) at the hip joint during the exercise.
	● Knee Flx – Increased knee flexion during the exercise.
	● Hip Flx – Increased hip flexion during the exercise.
Hip extension	● Hip ER – External rotation (ER) at the hip joint during the exercise.
	● Knee Flx – Increased knee flexion during the exercise.
	● Hip Abd – Increase hip abduction during the exercise.
Hip flexion	● Knee Flx – Increased knee flexion during the exercise.
Knee extension	● Hip Flx – Increased hip flexion during the exercise.
IRQ	● Hip Flx – Increased hip flexion during the exercise.
	● Hip ER – External rotation (ER) at the hip joint during the exercise.
SLR	● Knee Flx – Increased knee flexion during the exercise.

Following data recording and labeling, post analysis was performed using MATLAB (2012, The MathWorks, Natwick, USA). Six signals were obtained from each sensor for analysis; acceleration X, Y and Z, and gyroscope X, Y and Z. From these six signals, three additional signals were calculated; overall acceleration magnitude, pitch and roll. The nine available signals were then filtered using a 4^th^ order low-pass Butterworth filter with a cut-off frequency of 20 Hz. In order to train and test the classifier, the following features were then extracted from each of the available signals; signal mean, standard deviation, skewness, kurtosis, signal energy, level crossing rate, signal range, 25 percentile, 75 percentile and the variance of the wavelet coefficients using the Daubechies 5 mother wavelet to level 6.

Although each of these features could be useful to represent the data, it is not good practice to employ a large number of features when only a small number of trials are available as, by doing so, it is possible to over-fit the model, producing very good classification results during training but significantly poorer results during testing. In order to reduce the number of employed features principle component analysis (PCA) was performed [24]. PCA converts the set of features from a 126 dimensional matrix, with possibly correlated variables, into a set of principle components which are linearly uncorrelated. During analysis, the components which accounted for 99% of the variance were selected as the features. However, these new “features” no longer have any physical meaning (such as max, min etc.). It should also be noted that this process of feature selection using PCA is only performed on the training data, with the test data remaining unseen to the setup to refrain from biasing the system. The test data is reduced using the coefficients found using the training data.

In order to allow for multi-class analysis, in which the classifier attempts to specify which error occurred, the author selected the error from each trial which had the largest severity measure attributed with it. Similarly, any trials with errors which were deemed un-classifiable were also removed. An example of such a deviation is lateral trunk flexion during the hip flexion exercise. This deviation is un-classifiable as there were no sensors positioned above the thigh and so movement of the trunk cannot be detected. Following these steps, each trial had a corresponding class label which described the most severe error observed. It should also be noted that the exercise being performed was known *a priori* by the classifier. Therefore the classifier model did not have to determine which exercise was being performed but instead had to determine whether the known exercise was being performed correctly or not.

A logistic regression classifier was used to perform classification. Logistic regression is a discriminative probabilistic classification model that operates over real-valued vector inputs. The probabilities describing the possible outcomes of a single trial are modelled, as a function of the explanatory (predictor) variables, using a logistic function. Logistic regression therefore measures the relationship between a categorical dependent variable and one or more independent variables, which are usually (but not necessarily) continuous, by using probability scores (value between 0 and 1) as the predicted values of the dependent variable. The advantages of this classifier include its low complexity and its robustness against over-fitting compared to some other classification techniques [25]. During analysis several other types of classifiers were also tested including k-nearest neighbours, SVMs and naïve Bayes classifiers, however none were shown to provide significantly improved results on this dataset for the increased computational time required. For the multi-class classification problem a bank of one-versus-all classifiers were employed, where one of the possible deviations is set as the positive class and all other trials are set as the negative class. This was then repeated with each possible deviation set as the positive class. The final output class was chosen as the deviation which, when set as the positive class, resulted in the highest probability score. In the unlikely event of two deviations having the same probability score, the deviation which had the highest occurrence in the training set was chosen. Each classifier was trained and tested using leave-one-subject-out cross-validation (LOSOCV) and results were presented using the accuracy, sensitivity and specificity metrics. Accuracy measures the overall effectiveness of a classifier and is computed by taking the ratio of correctly classified examples and the total number of examples available. Sensitivity measures the effectiveness of a classifier at identifying a desired label, while specificity measures the classifiers ability to detect negative labels [19]. For each exercise, efficacy scores were calculated using the data from the foot, shin and thigh sensors individually as well as the various combinations of sensors (i.e. foot and shin, foot and thigh, shin and thigh, and foot, shin and thigh).

## Results

Fifty-eight participants (19 male, 39 female, age: 53.9 ± 8.5 years, height: 1.69 ± 0.08 m, weight: 74.3 ± 13.0 kg) took part in this investigation. The clinical information of the sample of participants is presented in Table 2. One study participant only performed the three exercises in lying due to time constraints, three subjects were not able to perform the SLR exercise and data were lost for one participant during the heel slide exercise and for another during the knee extension exercise due to sensor failure. This resulted in a total of 570 trials for the heel slide exercise and the three exercises in standing, 550 trials for the SLR and IRQ exercises, and 560 trials for the knee extension exercise.

**Table 2:** **Clinical information regarding the presenting condition of the study participants**

Condition	N
Osteoarthritis of the knee joint	14
Osteoarthritis of the hip joint	9
Osteoarthritis of the knee and hip joint	4
Post meniscectomy	3
Knee ligament injury	4
Instability of knee joint	4
Non-specific low back pain	18
Unknown	2

The results of the paper are presented in Table 3 and Table 4. Table 3 presents the efficacy scores obtained using binary classification (correct or incorrect) for each of the individual sensors as well as all combinations of sensors. Relatively high average efficacy scores were achieved using binary classification. Using three sensors, an average accuracy of 81%, sensitivity of 70%, and specificity of 70% were achieved at classifying an exercise as correctly or incorrectly performed. Using two sensors, an average accuracy of 82%, sensitivity of 83% and specificity of 70% were achieved, while using a single sensor achieved an average accuracy of 83%, sensitivity of 82%, and specificity of 72% at classifying an exercise as correctly or incorrectly performed.

**Table 3:** **Binary classification to identify an exercise as correct or incorrect**

	Sensor position						
	Foot	Shin	Thigh	Foot & shin	Shin & thigh	Thigh & foot	All three
**Heel slide**							
**Sensitivity (%)**	94	91	92	90	86	91	90
**Specificity (%)**	29	36	44	32	35	37	34
**Accuracy (%)**	76	76	79	73	72	75	74
**Hip abduction**							
**Sensitivity (%)**	66	71	77	63	61	71	61
**Specificity (%)**	91	95	96	93	94	87	94
**Accuracy (%)**	87	92	94	88	89	85	89
**Hip extension**							
**Sensitivity (%)**	96	84	93	84	84	98	84
**Specificity (%)**	99	100	100	97	100	99	100
**Accuracy (%)**	99	99	99	96	99	99	98
**Hip flexion**							
**Sensitivity (%)**	72	73	56	74	59	70	66
**Specificity (%)**	92	92	91	94	90	94	92
**Accuracy (%)**	88	88	83	90	84	89	86
**IRQ**							
**Sensitivity (%)**	86	90	92	91	91	88	90
**Specificity (%)**	20	28	40	31	37	41	44
**Accuracy (%)**	65	70	75	72	73	73	75
**Knee extension**							
**Sensitivity (%)**	85	86	90	85	86	89	90
**Specificity (%)**	36	34	67	38	57	55	57
**Accuracy (%)**	71	71	83	72	77	79	80
**SLR**							
**Sensitivity (%)**	64	58	72	64	71	77	69
**Specificity (%)**	64	58	68	62	69	74	68
**Accuracy (%)**	64	58	70	63	70	75	68
**Average across all seven exercises**							
**Sensitivity (%)**	80	79	82	79	77	83	79
**Specificity (%)**	62	63	72	64	69	70	70
**Accuracy (%)**	79	79	83	79	81	82	81

Mean Sensitivity, Specificity and Accuracy Scores for each exercise are presented.

**Table 4:** **Multi-label classification results obtained when trying to identify the error that occurred during an exercise**

	Sensor position						
	Foot	Shin	Thigh	Foot & shin	Shin & thigh	Thigh & foot	All three
**Heel slide**							
**Sensitivity (%)**	35	37	42	37	35	33	39
**Specificity (%)**	80	81	83	80	80	80	80
**Accuracy (%)**	71	71	72	69	67	68	68
**Hip abduction**							
**Sensitivity (%)**	37	43	42	35	39	41	39
**Specificity (%)**	78	82	80	79	80	80	81
**Accuracy (%)**	45	53	53	43	50	49	49
**Hip extension**							
**Sensitivity (%)**	69	66	61	50	47	49	48
**Specificity (%)**	75	75	69	76	73	72	75
**Accuracy (%)**	60	60	50	61	56	55	60
**IRQ**							
**Sensitivity (%)**	38	42	50	45	48	51	53
**Specificity (%)**	69	72	77	74	76	77	78
**Accuracy (%)**	63	67	73	70	72	72	73
**Average across all four exercises**							
**Sensitivity (%)**	45	47	49	42	42	44	45
**Specificity (%)**	76	78	77	77	77	77	79
**Accuracy (%)**	60	63	61	61	61	61	63

Mean Sensitivity, Specificity and Accuracy Scores for each exercise are presented.

Table 4 outlines the results using multi-label classifiers. Multi-label classifiers were not employed for the hip flexion, knee extension or SLR exercise, as only one error label was studied for these exercises and therefore binary classification was sufficient to quantify the data obtained during these exercises. Moderate efficacy scores were achieved using multi-label classification. Using three sensors, an average accuracy of 63%, sensitivity of 45%, and specificity of 79% were achieved at correctly classifying the error that had occurred. Using two sensors, an average accuracy of 61%, sensitivity of 44% and specificity of 77% were achieved, while using a single sensor achieved an average accuracy of 63%, sensitivity of 49%, and specificity of 77% at correctly classifying the error that had occurred.

## Discussion

This research has explored whether lower limb exercise performance can be classified in a cohort of patients using data from inertial sensors and machine learning classification methods. The results obtained revealed that it is possible to correctly classify lower limb exercise performance using inertial sensors with satisfactory levels of accuracy. In addition the results revealed that reducing the number of sensors used, from three to one, does not appreciably reduce the accuracy of this method of classifying exercise performance. In fact, for some exercises, a single sensor approach is more accurate at correctly classifying exercise performance, than two or three sensor on the lower limb. One possible reason for this anomaly may be that the increase in the data available, due to the increased number of sensors utilized, causes the model to over fit the training data thus causing poorer results on the test data. Also, for many of the exercises, one sensor is sufficient to provide information regarding the movement or deviation. The addition of a second, or third, sensor does not provide sufficient additional information and thus can introduce additional noise into the system. Future advances of the employed technique may aid to alleviate these problems. These results provide further evidence to support the use of a single inertial sensor as an input to an exercise biofeedback system.

Machine learning classification techniques were used to quantify the inertial sensor data acquired during the seven exercises studied. These classifier techniques allow for real-time, objective quantification of data, which is essential in an intelligent exercise biofeedback system. Binary classifiers (correct or incorrect) and multi-label classifiers (which determine which error in a set of errors) were employed and the efficacy of these classifiers were quantified using three efficacy scores; accuracy, sensitivity, and specificity. Binary classification of the inertial sensor data revealed that it is possible to classify performance of an exercise as correct or incorrect with relatively high accuracy, sensitivity and specificity. The average scores across all seven exercises revealed that a single sensor on the thigh is the best approach to identify whether an exercise is performed correctly or incorrectly, achieving higher efficacy scores than the various two sensor approaches or all three sensors together. Analysing each exercise individually, a single sensor on the thigh yields the best efficacy scores for classifying five of the seven exercises studied as correct or incorrect (heel slide, hip abduction, hip extension, IRQ, and knee extension exercise). Two sensors (thigh and foot) give the best efficacy scores for classifying the SLR exercise as correctly or incorrectly performed. However, reducing to a single sensor on the thigh did not markedly change the efficacy scores (5% difference in accuracy, 5% in sensitivity, and 6% in specificity). Likewise for the hip flexion exercise, two sensors (foot and shin) gave the best efficacy scores at classifying correct or incorrect exercise performance, however reducing to a single sensor, this time on the shin, did not exceptionally decrease the efficacy scores (2% decrease in accuracy, 1% in sensitivity, and 2% in specificity).

A significant feature of an exercise biofeedback system is for it to be able to identify when an exercise is being performed correctly or incorrectly. The system needs also to be able to detect and recognise the error that has occurred, to give effective feedback on performance to the user. Therefore, multi-label classifiers were applied to the data acquired in this study in an attempt to classify the error that had occurred. As multi-label classifiers were only required when more than one error had occurred, the knee extension, SLR and the hip flexion exercises were not quantified using multi-label classifiers. Multi-label classification of the inertial sensor data revealed that it is possible to identify the error that had occurred with moderate accuracy, sensitivity and specificity. The average scores across all four exercises revealed that a single sensor on the thigh is the best approach to identify the error that had occurred. Analysing each exercise individually, a single sensor on the thigh yields the best efficacy scores for identify the errors during the heel slide exercise. For the hip abduction exercise a single sensor on the shin yielded the best efficacy scores, while a single sensor on the foot yielded the best scores for the hip extension exercise. For the IRQ exercise, while all three sensors achieve the best efficacy scores, a single sensor on the thigh achieved comparable classification efficacy scores at identifying the errors that occur (accuracy scores equal, 3% difference in sensitivity, and 1% difference in specificity).

Comparing the results of the binary and multi-label classifier, there is an appreciable drop in the accuracy scores for the hip abduction (40% for three sensors, 19% for two sensors, and 41% for one sensor) and the hip extension (38% for three sensors, 41% for two sensors, and 43% for one sensor) exercise. The accuracy scores also drops for the heel slide (9% for three sensors, 5% for two sensors and one sensor) and the IRQ (2% for three, two and one sensor) exercise, however the difference is not as large. A reduction in accuracy scores is expected when comparing the binary and multi-label classification scores, as it is a more complex task to identify the range of errors that may occur during the exercise. However, an average accuracy score of at least 60% was achieved across all exercises using multi-label classification methods (when using data from three sensors, the various combinations of two sensors, or from a single sensor).

The results obtained in this study are comparable to previous research that has evaluated the use of inertial sensors and machine learning methods to quantify exercise performance [19, 20]. The ability of the classifier presented in this study to detect when an exercise is performed incorrectly is comparable to that presented in [19]. However less favourable results were obtained in this current study when the multi-label classifiers were employed as compared to that presented in [20]. However this is as expected as LOSOCV was used in this study as compared to the 10-fold cross validation that was performed in [20]. In addition, a balanced dataset were used in [20], where there was an equal number of error labels as correct labels. Using a balanced dataset helps to improve the efficacy scores obtained but in an unrealistic manner. Another possible explanation for the lower efficacy scores in this study is the variations in the way different subjects produce the same errors. Though two patients may produce the same error in exercise performance, each may have committed the error in a different way. Nevertheless, the results obtained in this study are important as they provide further evidence to suggest that a single sensor can provide sufficient information on exercise performance, and therefore can be used as a viable input to an exercise biofeedback system. A single sensor approach is desirable as not only does it reduce the cost of the system but also avoids cumbersome set up and calibration procedures.

These findings therefore prompt the development of a simple biofeedback system using a single inertial sensor to monitor exercise performance in the home. By developing a system that can provide biomechanical feedback to patients, it is hoped that patients will perform their exercises at home more accurately. In addition, it is hoped that patient motivation to perform exercises will be increased, which may enhance rehabilitation and recovery.

There are a number of limitations to this study which need to be considered. Firstly, the data gathered in this study were gathered in a clinical environment, where the exercises were performed under controlled conditions. In this study, participants performed the exercises wearing appropriate clothing for exercise, and in a clutter free environment. These conditions may differ from what may occur in reality in the home. Furthermore, the errors in exercise performance observed in this study differ from what is reported as commonly occurring deviations for these exercises (26). In reality, further deviations in performance may occur if a larger sample or a different population were studied or if a different study environment were used. Another limitation of this study is that the sample selected was not a homogenous group. The sample of participants taking part in this study were attending the physiotherapy clinic for rehabilitation for a multitude of musculoskeletal conditions. As the sample was not large enough, it was not possible to sub-divide the sample into groups to investigate whether the classifier performed differently for different populations. While the results cannot be generalized to a specific population, the heterogeneity of the sample increases the external validity of the results.

The complex dataset also provided limitations for the study. As the participants’ movements were not corrected during each trial, the datasets for the various exercises were often highly unbalanced. For example, one exercise could have 50% more correct trials than incorrect. The examination of the multi-label classifier would then further exacerbate this unwanted data unbalancing to the point where some deviations would have too few trials to allow for classification. At least forty examples of each label were required to train and test the model sufficiently, so trials that had error labels with less than forty examples were excluded. The number forty was empirically chosen to ensure that there was always sufficient data available and therefore ensure that a deviation did not occur in the test case which had not been seen previously in the training of the model. This unbalanced nature of the datasets caused some of the exercises to present lower sensitivity or specificity scores than the more naturally balanced datasets. It should be again noted that this work only seeks to provide evidence to support the use of single inertial sensors as an input to an exercise biofeedback system. Further work is required to develop the system and improve the overall classification results to levels acceptable for use in rehabilitation. A further limitation is that, for each exercise, only the error labels with the largest severity attributed to it were included in the analysis. Failing this simplification, the number of possible classes would grow exponentially with the number of possible deviations. Preliminary evaluations also suggested that error labels that involved contralateral limb or upper body movements could not be classified. For this reason, any trials with errors that were deemed un-classifiable (e.g. trunk flexion) were removed. Future work should endeavour to overcome the data unbalancing problem and examine and classify all potential errors, which will be important as researchers develop inertial sensor based exercise biofeedback systems.

## Conclusion

Poor adherence and poor performance of rehabilitation exercises motivates the need for exercise biofeedback systems to monitor the performance of rehabilitation exercises in the home. Inertial sensors have previously been advocated due to their relative low expense and the fact that they can be readily deployed in the home. Current systems using inertial sensors rely on multiple sensors to track movement and evaluate performance during lower limb exercises. However, using multiple sensors increases both the cost and the setup time required and thus reduces the likelihood of full patient adherence. This research therefore sought to answer two separate research questions: 1) whether inertial sensors can classify exercise performance in patients performing lower limb exercises and thus be used in the development of an exercise biofeedback system, and 2) whether a reduction in the number of sensors employed in the feedback system would have a significant detrimental effect on the efficacy results.

Machine learning classification methods were applied to quantify the data gathered in this study and the results revealed that it is possible to distinguish between correct and incorrect performance of an exercise using three, two, or one sensor(s), with an average accuracy of 81%, 82%, and 83% respectively. The results also revealed that it is possible to not only identify that the movement was performed incorrectly but, more specifically, which error was performed within each exercise. Moderate average efficacy scores were obtained across all seven exercises (average accuracy = 63% for three sensors, 61% for two sensors, and 63% for one sensor), however for some exercises the efficacy scores achieved were low. Nevertheless these results provide an answer to our first research question above in that inertial sensors can indeed be used to classify exercise performance in patients performing lower limb exercises. Further work is required to improve the accuracy and robustness of the system prior to implementing in a feasible biofeedback system.

This work also sought to determine whether a single sensor placed on the lower limb can provide sufficient information to classify performance. Reducing the number of sensors, from three to one was found to not have a considerable impact on the accuracy of the proposed technique, and in some cases, a single sensor performs better at evaluating exercise performance than the various combinations of two and three sensors. These results allow for the development of an inertial sensor based biofeedback platform, which aims to guide and enhance rehabilitation exercise performance in the home.

## Authors’ original submitted files for images

Below are the links to the authors’ original submitted files for images. Authors’ original file for figure 1

## Abbreviations

OA: Osteoarthritis
RGB: Red-Green-Blue
3D: Three-dimensional
SLR: Straight leg raise
IRQ: Inner range quadriceps
9DOF: 9 degrees of freedom
PCA: Principle Component Analysis
LOSOCV: Leave-one-subject-out-cross-validation.
