# Supplementary figures and images for: Rehabilitation exercise assessment using inertial sensors: a cross-sectional analytical study
Source: J Neuroeng Rehabil. 2014 Nov 27;11:158. doi: 10.1186/1743-0003-11-158 (PMC4280766; doi:10.1186/1743-0003-11-158)

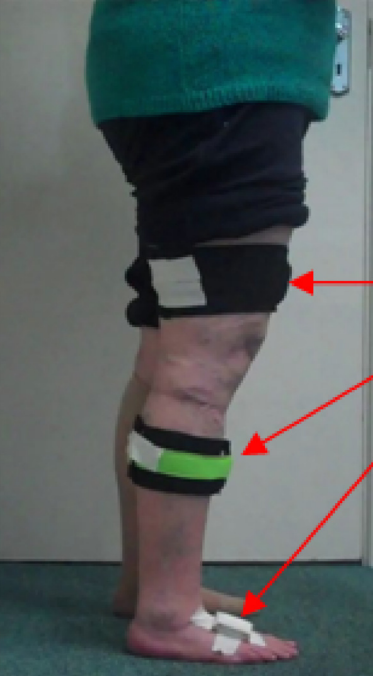

**Inertial Sensor on Thigh, Shin,  
and Foot**

Supplement: Supplementary file 1 — Authors’ original file for figure 1 [file 12984_2014_687_MOESM1_ESM.pdf]
